# ADC-Based Stratification of Molecular Glioma Subtypes Using High b-Value Diffusion-Weighted Imaging

**DOI:** 10.3390/jcm10163451

**Published:** 2021-08-04

**Authors:** Nils C. Nuessle, Felix Behling, Ghazaleh Tabatabai, Salvador Castaneda Vega, Jens Schittenhelm, Ulrike Ernemann, Uwe Klose, Johann-Martin Hempel

**Affiliations:** 1 Department of Neuroradiology, University Hospital Tübingen, Eberhard Karls University, 72076 Tübingen, Germany; ulrike.ernemann@med.uni-tuebingen.de (U.E.); uwe.klose@med.uni-tuebingen.de (U.K.); johann-martin.hempel@med.uni-tuebingen.de (J.-M.H.); 2 Department of Neurosurgery, University Hospital Tübingen, Eberhard Karls University, 72076 Tübingen, Germany; felix.behling@med.uni-tuebingen.de; 3 Departments of Neurology and Interdisciplinary Neuro-Oncology, University Hospital Tübingen, Hertie Institute for Clinical Brain Research, Eberhard Karls University, 72076 Tübingen, Germany; ghazaleh.tabatabai@med.uni-tuebingen.de; 4 Werner Siemens Imaging Center, Department of Preclinical Imaging and Radiopharmacy, University Hospital Tübingen, Eberhard Karls University, 72076 Tübingen, Germany; salvador.castaneda@med.uni-tuebingen.de; 5 Department of Pathology and Neuropathology, University Hospital Tübingen, Institute of Neuropathology, Eberhard Karls University, 72076 Tübingen, Germany; jens.schittenhelm@med.uni-tuebingen.de

**Keywords:** diffusion-weighted imaging, DWI, diffusion kurtosis imaging, DKI, ADC, glioma

## Abstract

Purpose: To investigate the diagnostic performance of in vivo ADC-based stratification of integrated molecular glioma grades. Materials and methods: Ninety-seven patients with histopathologically confirmed glioma were evaluated retrospectively. All patients underwent pre-interventional MRI-examination including diffusion-weighted imaging (DWI) with implemented b-values of 500, 1000, 1500, 2000, and 2500 s/mm^2^. Apparent Diffusion Coefficient (ADC), Mean Kurtosis (MK), and Mean Diffusivity (MD) maps were generated. The average values were compared among the molecular glioma subgroups of IDH-mutant and IDH-wildtype astrocytoma, and 1p/19q-codeleted oligodendroglioma. One-way ANOVA with post-hoc Games-Howell correction compared average ADC, MD, and MK values between molecular glioma groups. A Receiver Operating Characteristic (ROC) analysis determined the area under the curve (AUC). Results: Two b-value-dependent ADC-based evaluations presented statistically significant differences between the three molecular glioma sub-groups (*p* < 0.001, respectively). Conclusions: High-b-value ADC from preoperative DWI may be used to stratify integrated molecular glioma subgroups and save time compared to diffusion kurtosis imaging. Higher b-values of up to 2500 s/mm^2^ may present an important step towards increasing diagnostic accuracy compared to standard DWI protocol.

## 1. Introduction

Gliomas are one of the most common primary central nervous system tumors and are, in most cases, associated with poor overall survival [1]. Treatment options include surgical resection, chemotherapy, and radiation therapy, depending on the histopathological entity [1,2].

Unfortunately, the distinction between different glioma subtypes with sufficient sensitivity and specificity remains challenging in preoperative settings and imaging. A reliable pre-interventional glioma stratification based on the expected molecular glioma profile may impact therapeutic options, the extent of planned surgical resection, and prognosis [3,4,5].

Various models have been proposed in previous reports to distinguish non-invasively different tumor entities using MRI. ADC-map-based tumor evaluation from diffusion-weighted imaging (DWI) seems to be a promising means of differentiation [6,7].

Standardized MRI protocols for glioma patients have been proposed. They are used in most centers, but standardized evaluation methods for non-invasive grading and follow-up have not yet been implemented in the clinical workflow [8,9].

In the past, diffusion kurtosis imaging (DKI) and high-b-value DWI showed great potential and good diagnostic capability. For DKI, several multidirectional b-values are needed. They are correlated with an extended acquisition time and complex post-processing [10,11].

However, the calculation of ADC maps from DWI is a fast and straightforward procedure that has already shown the potential for distinguishing high-grade from low-grade glioma according to the previous 2007 World Health Organization Classification of Tumors of the Central Nervous System (2007 CNS WHO) [6]. Therefore, this study aims to evaluate high-b-value ADC-based tumor classification’s diagnostic performance and compare it with DKI-based tumor stratification according to integrated glioma grades.

## 2. Materials and Methods

### 2.1. Study Types and Ethics

This study is a retrospective analysis of prospective data acquired in a single-center, non-randomized trial, approved by the local institutional review board of the University Hospital Tuebingen (Ref. No. 727/2017BO2). The trial was conducted based on the International Conference on Harmonization: Good Clinical Practice guidelines and according to the revised version of the declaration of Helsinki. All patients provided written informed consent for the imaging surveys and the subsequent use of images for scientific and research purposes.

### 2.2. Patient Selection and Stratification

**Figure 1:**
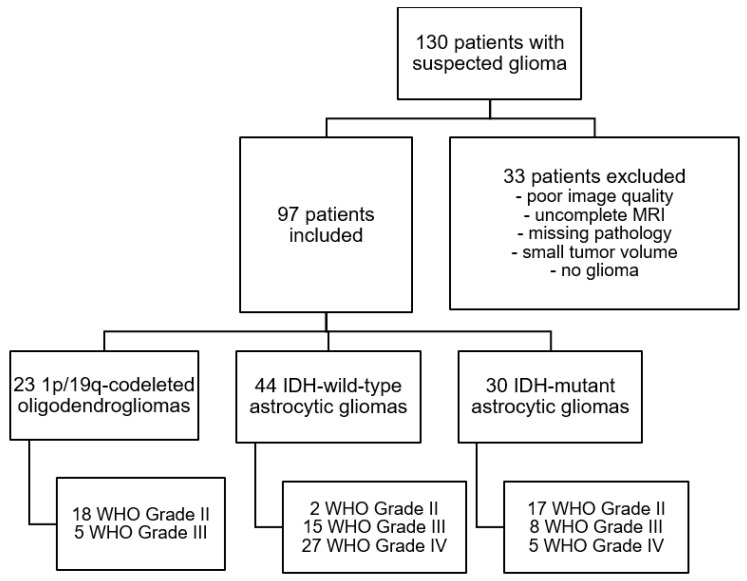
Patient selection algorithm according to the STROBE guidelines.

The study cohort was selected from 130 patients suspected to have a primary CNS tumor between October 2012 and September 2017. Seventy-seven of the patients had been assessed previously with diffusion kurtosis imaging [12]. All patients received preoperative cerebral MR scans within two weeks of diagnosis, and none of them were receiving steroid therapy at the time of analysis. Thirty-three patients were removed from the study collective because of low image quality (e.g., moving artifacts, early stop to the MR examination), non-existing histopathological sampling, infectious diseases, gliosis, or minimal tumor volume. The final study cohort comprised 97 patients with a mean age of 51.6 ± 15.3 years (see Figure 1).

### 2.3. Glioma Classification

The final glioma classification was based on the current 2016 CNS WHO criteria [4] and included histopathological and molecular data. 

The IDH mutation status was assessed by immunohistochemistry with a mutation-specific IDH1 R132H antibody [13]. This was followed by Sanger sequencing of the negative cases to detect any non-canonical IDH1/2 mutations [14]. Nuclear ATRX status in tumor cells was determined by immunohistochemistry, as described previously [15]. A synthetic high-resolution microsatellite PCR gel was used to study chromosome 1p/19q LOH in all tumors with an oligodendroglial component [16].

In the integrated approach, the combination of the loss of ATRX expression and the presence of IDH1/2 mutation characterized IDH-mutant (IDH-mut) astrocytoma, including its most aggressive histological subtype of Astrocytoma, IDH-mutant, WHO grade 4 according to the cIMPACT-NOW update 5 [11]. These tumors were formerly designated as Glioblastoma, IDH mutant, WHO grade IV. Grade IV tumors with IDH wild-type status and retained ATRX expression are primary Glioblastomas (IDH-wt). Oligodendrogliomas were defined by synchronous 1p/19q loss of heterozygosity and IDH1/2 mutation (1p/19q-Codel Oligodendroglioma), and they are typically associated with maintained ATRX expression (see Figure 1) [17,18,19,20].

The final groups consisted of 23 1p/19q-Codel Oligodendrogliomas, 44 IDH wild-type Astrozytomas, and 30 IDH-mutant Astrozytomas (17 WHO Grade II, 8 WHO Grade III, 5 WHO Grade IV).

### 2.4. MR Imaging

MRI was performed using a 3.0 T MRI scanner (Biograph mMR, Siemens Healthineers, Erlangen, Germany). Imaging was carried out using a transversal 2D-encoded T2-weighted fluid-attenuated inversion recovery (FLAIR) sequence (TR/TE, 9000/87 ms; inversion time, 2500 ms; 36 slices; slice thickness, 4.0 mm) and a sagittal 3D-encoded isotropic magnetization prepared rapid acquisition gradient echo (MPRAGE) sequence (TR/TE, 1900/2.4 ms; TI, 900 ms; 124 partitions; slice thickness, 1.0 mm) before and after contrast medium administration (0.1 mL/kg body weight gadobutrol) were part of the conventional MR examination protocol.

The Spin-echo echo-planar imaging DWI sequence used b values of 0, 500, 1000, 1500, 2000, and 2500 s/mm^2^ with encoding in 6 directions. The other imaging parameters were as follows: TR 5900 ms, TE 95 ms; field of view, 250 × 250 mm^2^; matrix 128 × 128; 25 slices; slice thickness, 5 mm; bandwidth, 965 Hz/pixel; parallel imaging with a sensitivity encoding factor of two in the anteroposterior direction.

### 2.5. Image Post-Processing

Imaging post-processing used Matlab (MatLab 9.2, Natwick, MA, USA).

All six measured directions of the six b-values in DWI were averaged.

Five different sets of apparent diffusion coefficient (ADC) maps were calculated using the b-value of 0 s/mm^2^ as a reference baseline value (B_0_ADC maps) and one other b-value (B_0/500_ADC, B_0/1000_ADC, B_0/1500_ADC, B_0/2000_ADC, and B_0/2500_ADC).

Another four sets of ADC-maps were calculated with a baseline b-value of 500 s/mm^2^ (B_500_ADC maps) and one other b-value (B_500/1000_ADC, B_500/1500_ADC, B_500/2000_ADC, and B_500/2500_ADC) to avoid perfusion-based influence on images and perfusion-artifacts.

Additionally, mean kurtosis (MK) and mean diffusivity (MD) maps were calculated, one time using all b-values, including 0 s/mm^2^ (MK_0_ and MD_0_), and a second time excluding the b-value of 0 s/mm^2^ (MK_500_ and MD_500_), as described in previous studies, to compare them to the new procedure of ADC-map-based evaluation [10,11,21].

In total, 14 different sets of ADC (*n* = 9), MK (*n* = 2), and MD (*n* = 2) parametric maps were generated for further analysis.

Subsequently, all maps were registered and interpolated to the matrix of the FLAIR images.

The volumes of interest (VOIs) were manually delineated on the FLAIR sequences based on T2 signal alterations. The VOIs were delineated around the entire tumor volume on multiple slices to minimize sampling bias. Tumor necrosis and surrounding edema and great vessels were excluded from the VOIs.

**Figure 2:**
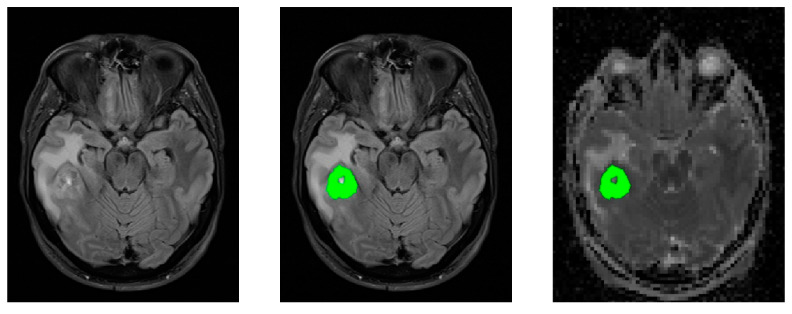
FLAIR and DW Images of histopathologically confirmed IDH-wt Glioblastoma-patient: unmodified FLAIR (**left**); FLAIR with drawn-in tumor region (**green**); excluding central necrosis and surrounding edema (**middle**); ADC map from DWI, fitted to the matrix of the FLAIR images, with transferred tumor region (**right**).

The VOIs were then transferred from the FLAIR images to the ADC, MK, and MD maps. Average ADC, MK, and MD values, as well as standard deviation, were calculated for each tumor region. This process is visualized in Figure 2. Subsequently, statistical differences were calculated, and a receiver operating characteristics (ROC) analysis was performed.

## 3. Results

### 3.1. ADC

Average ADC (avADC) values were significantly higher in IDH-mut gliomas than in oligodendrogliomas and IDH-wt gliomas. AvADC values in oligodendrogliomas were significantly higher than in IDH-wt gliomas. These effects were found in all comparisons and are presented in Table 1.

Higher b-values provided higher levels of significant differences between the three glioma-subtypes, even if the signal-to-noise ratio was higher for low b-values.

In the five B_0_ADC maps, differentiation between the three glioma subtypes was at the highest significance level when using the highest b-value of 2500 s/mm^2^ (see Table 2).

The avADC values based on the four additional B_500_ADC maps showed the same relations as the B_0_ADC-maps between the three tumor groups (see Table 3).

Compared with the avADC values based on B_0_ADC maps, they showed a much better correlations with the tumor entities in lower b-values and slightly better results in terms of their high b-values such as 2500 s/mm^2^ (see Table 4).

**Figure A1:**
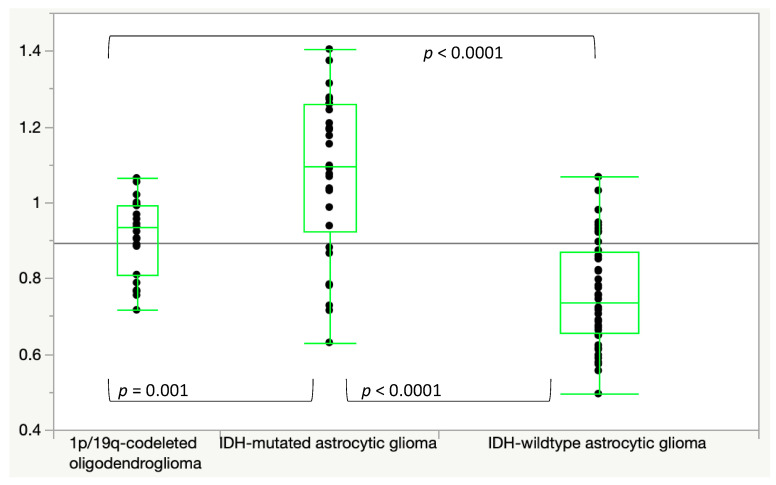
Distribution of the average ADC values using b-values of 500 s/mm^2^ and 2500 s/mm^2^, classified into the three molecular glioma groups: ADC, apparent diffusion coefficient.

The distribution of avADC values can be seen in Figure A1.

### 3.2. MK

MK-map-based evaluation of the tumor regions revealed the highest level of significant differences between the different glioma subtypes.

All three groups showed highly significant differences when including all measured b-values (see Table 5 and Table 6).

**Figure A2:**
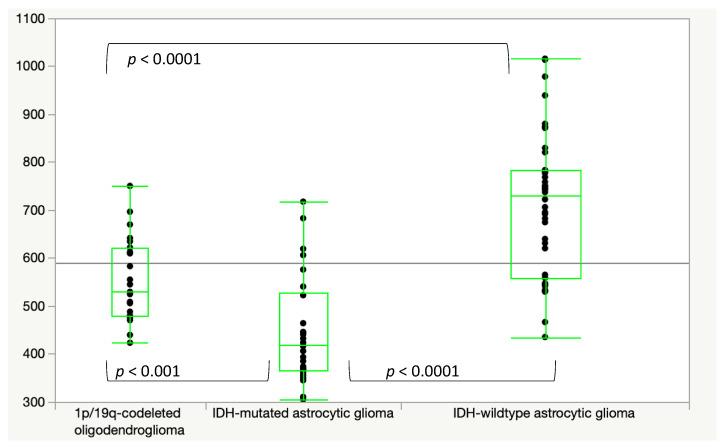
Distribution of the mean kurtosis values using all measured b values from 0 s/mm^2^ to 2500 s/mm^2^, classified into the three molecular glioma groups.

The distribution of MK values of each patient in one MK map can be seen in Figure A2.

### 3.3. MD

The evaluation of MD maps displayed differences between the three groups, as well. Calculation of the MD maps excluding the b-value of 0 s/mm^2^ resulted in better discrimination, as displayed in Table 6.

Nevertheless, the level of significance was lower than in the two other evaluation procedures described.

The comparison between 1p/19q-Codel Oligodendroglioma and IDH-wt astrocytic gliomas in the MD-map, including the b-value of 0 s/mm^2^, showed no significant difference (see Table 6).

## 4. Discussion

The aim of this study was to evaluate a high-b-value ADC-based tumor classification’s diagnostic performance and compare it with DKI-based tumor stratification according to the latest integrated glioma grades. In contrast to previous studies, not only were multiple b-value-dependent MK and MD analyses used, but a two b-value-dependent ADC-map-based method was also performed [5,12,22,23]. High-b-value ADC from preoperative DWI may be used to stratify molecular glioma subgroups and save time compared to DKI. Higher b-values up to 2500 s/mm^2^ may increase diagnostic accuracy compared to the standard DWI protocol.

Diffusion imaging parameters enable a quantitative assessment of water diffusion behavior in the brain. However, the water diffusion probability distribution is influenced by diffusion barriers. Thus, ADC from DWI, as well as MK and MD from DKI, may reflect a tissue’s heterogeneity, complexity, and micro-structure [24,25].

In the literature, IDH-mut astrocytic gliomas with a more homogeneous and looser cell composition show lower MK and higher MD and ADC values than IDH-wt gliomas with increased MK and decreased ADC and MD values due to increased cellularity, cellular heterogeneity, hemorrhage, necrosis, and microvascular proliferation [11,26]. 1p/19q-Codel Oligodendrogliomas also have higher MK and lower ADC and MD values than IDH-mut astrocytic gliomas because of their higher tumor cellularity and mitotic activity [11].

Our results may underline the hypothesis that different molecular glioma subtypes seem to show differences in diffusion-weighted MR-imaging. Specifically, higher b-values presented higher significance levels and might lead to better results in differentiating the three molecular glioma groups. Subsequently, a high b-value DWI could be a promising step towards non-invasive pre-interventional classification. In contrast, other publications clarified that distinction based on pre-interventional low b-value diffusion-weighted MR-imaging into the molecular subgroups might also be applicable [27].

However, the differences between the three tumor subgroups are statistically significant, and the overlapping presents a considerable limitation for clinical use or decision making.

Higher b-values demonstrated higher diagnostic accuracy but were not sufficiently evaluated in this context [28]. As, in this study, *p*-values improved with higher b-values, further research is needed to assess the potential of ultra-high b-values up to 5000 s/mm^2^ in distinguishing different types of gliomas. Looking at the high b-values, the acquisition becomes very time-consuming, as many averages or measured directions are required to get an acceptable signal-to-noise ratio, making the protocol more vulnerable to movement artifacts. ADC maps in this study only required two b-values instead of the six needed for DKI and led to comparable results. This reduction in the acquisition time by 66%, while showing comparable results, has a higher chance of being implemented in a routine clinical workflow.

The post-processing for ADC measurements is more straightforward than DKI, as most scanners provide ADC calculations by default. Previous studies demonstrated that DWI-based glioma classification into the two groups of high-grade (HGG) and low-grade glioma (LGG) was possible with a sensitivity of over 90% [6,29]. Unfortunately, most of these reports have a relatively small patient cohort [30]. In addition, the molecular tumor stratification, used in the present article, correlates better with the clinical outcome than the outdated 2007 CNS WHO classification in HGG and LGG, used by most other research groups, and subsequently enables support of the clinical decision-making process [6,7,29].

As performed in the present study, classification into molecular glioma subtypes has become an integral part of the current 2016 CNS WHO due to its prognostic importance [17,18,19]. Previous studies focusing on the differentiation between HGG and LGG do not consider these clinically and prognostically relevant features in glioma. As recent research focuses on monitoring patients post-interventionally via DWI and distinguishing recurrent glioma from pseudoprogression, the performance of ADC-based evaluation strategies in this context has great potential but needs further investigation [31].

The estimates from DWI and DKI are biased by micro-capillary perfusion through the intravoxel incoherent motion (IVIM) effect, especially in lower b-values from 0 to 300 s/mm^2^ [25,32,33]. However, the perfusion-based influence in DWI and DKI needs to be considered in differentiating glioma subtypes. B_500_ADC maps showed better results than the B_0_ADC maps, which may be explained by the perfusion influence described to impair DWI at lower b-values [10,34,35]. Different types of perfusion imaging, such as arterial spin labeling and dynamic contrast-enhanced perfusion, have been described in recent studies as a potential approach to grading and determining IDH-mutation status [21,36]. Comparing the different evaluation strategies in this study, the results provided slightly higher levels of significance in the kurtosis-based evaluation. The differences in the average values showed better significance levels between the three tumor groups than the two b-value-dependent ADC-based methods. This confirms the results of previous studies [11].

However, ADC-based results remained comparable despite the acquisition time for DKI being three times longer and, therefore, the evaluation includes three times more data than the ADC-based approach. The MD results were not better than those of the ADC maps and required the same acquisition time as the DKI.

### Limitations

This study is limited by its retrospective study design and different tumor locations. Additionally, the process of VOI delineation may have been subject to sampling bias because glioma infiltration may extend beyond T2 signal abnormalities [37,38]. The manual delineation of tumor volumes may risk possible bias, which could be reduced by automatic segmentation algorithms. However, studies have shown that the difference in tumor delimitation among different observers has a minor impact regarding the large number of voxels included in the histogram analysis [39,40]. There is potential bias regarding the relatively small numbers of patients with IDH_mut_ astrocytoma WHO grade 4 and IDH_wt_ AS2 (early precursor lesion of IDH_wt_ GBM). However, these numbers represent their natural incidence [17,18,19,20,41].

## 5. Conclusions

High-b-value ADC from preoperative DWI may be used to stratify molecular glioma subgroups and save time compared to DKI. Higher b-values of up to 2500 s/mm^2^ may increase diagnostic accuracy compared to the standard DWI protocol.

## Figures and Tables

**Table 1:** B_0_-avADC values of the three glioma subgroups (10^−3^ × mm^2^/s).

	IDH-mut Astrocytic Gliomas	1p/19q-Codel Oligodendroglioma	IDH-wt Astrocytic Gliomas
B_0/500_ADC	1.59 ± 0.26	1.42 ± 0.27	1.19 ± 0.2
B_0/1000_ADC	1.5 ± 0.27	1.32 ± 0.23	1.09 ± 0.19
B_0/1500_ADC	1.4 ± 0.26	1.21 ± 0.19	1.00 ± 0.18
B_0/2000_ADC	1.29 ± 0.24	1.11 ± 0.15	0.92 ± 0.16
B_0/2500_ADC	1.18 ± 0.22	1.01 ± 0.12	0.84 ± 0.14

**Table 2:** Statistical differences (*p*-values) and AUC from ROC analysis of the three glioma subgroups for B_0_ADC-evaluation: AUC, area under the curve; ROC, receiver operation characteristic; MK, mean kurtosis; MD, mean diffusivity.

	IDH-mut vs. IDH-wt Astrocytic Gliomas	IDH-mut vs. IDH-wt Astrocytic Gliomas; AUC	1p/19q-Codel Oligodendroglioma vs. IDH-wt Astrocytic Gliomas	1p/19q-Codel Oligodendroglioma vs. IDH-wt Astrocytic Gliomas; AUC	1p/19q-Codel Oligodendroglioma vs. IDH-mut Astrocytic Gliomas	1p/19q-Codel Oligodendroglioma vs. IDH-mut Astrocytic Gliomas; AUC
B_0/500_ADC	<0.0001	0.874	0.002	0.778	0.08	0.696
B_0/1000_ADC	<0.0001	0.883	<0.001	0.784	0.03	0.699
B_0/1500_ADC	<0.0001	0.879	0.0001	0.789	0.01	0.736
B_0/2000_ADC	<0.0001	0.883	<0.0001	0.795	0.005	0.747
B_0/2500_ADC	<0.0001	0.888	<0.0001	0.799	0.003	0.751

**Table 3:** B_500_-average ADC values of the three glioma subgroups (10^−3^ × mm^2^/s).

	IDH-mut vs. IDH-wt Astrocytic Gliomas	1p/19q-Codel Oligodendroglioma vs. IDH-wt Astrocytic Gliomas	1p/19q-Codel Oligodendroglioma vs. IDH-mut Astrocytic Gliomas
B_500/1000_ADC	1.41 ± 0.28	1.21 ± 0.19	0.99 ± 0.18
B_500/1500_ADC	1.31 ± 0.27	1.10 ± 0.15	0.90 ± 0.17
B_500/2000_ADC	1.19 ± 0.24	1.00 ± 0.12	0.83 ± 0.15
B_500/2500_ADC	1.07 ± 0.21	0.91 ± 0.10	0.76 ± 0.13

**Table 4:** Statistical differences (*p*-values) and AUC from ROC analysis of the three glioma subgroups for B_0_ADC evaluation: AUC, area under the curve; ROC, receiver operation characteristic; MK, mean kurtosis; MD, mean diffusivity.

	IDH-mut vs. IDH-wt Astrocytic Gliomas	IDH-mut vs. IDH-wt Astrocytic Gliomas; AUC	1p/19q-Codel Oligodendroglioma vs. IDH-wt Astrocytic Gliomas	1p/19q-Codel Oligodendroglioma vs. IDH-wt Astrocytic Gliomas; AUC	1p/19q-Codel Oligodendroglioma vs. IDH-mut Astrocytic Gliomas	1p/19q-Codel Oligodendroglioma vs. IDH-mut Astrocytic Gliomas; AUC
B_500/1000_ADC	<0.0001	0.878	<0.0001	0.798	0.01	0.723
B_500/1500_ADC	<0.0001	0.880	<0.0001	0.795	0.003	0.746
B_500/2000_ADC	<0.0001	0.883	<0.0001	0.806	0.002	0.751
B_500/2500_ADC	<0.0001	0.884	<0.0001	0.808	0.001	0.751

**Table 5:** MK and MD values of the three glioma subgroups (MK metrics are dimensionless, MD in 10^−6^ × mm^2^/s).

	IDH-mut Astrocytic Gliomas	1p/19q-Codel Oligodendroglioma	IDH-wt Astrocytic Gliomas
MK_0_	446.1 ± 109	552.8 ± 88	705.1 ± 145
MK_500_	593.8 ± 121	694.4 ± 114	825.3 ± 145
MD_0_	1681 ± 293	1504 ± 313	1224 ± 257
MD_500_	1518 ± 314	1288 ± 227	1029 ± 224

**Table 6:** Statistical differences (*p*-values) and AUC from ROC analysis of the three glioma subgroups for MK and MD evaluation: AUC, area under the curve; ROC, receiver operation characteristic; MK, mean kurtosis; MD, mean diffusivity.

	IDH-mut vs. IDH-wt Astrocytic Gliomas	IDH-mut vs. IDH-wt Astrocytic Gliomas; AUC	1p/19q-Codel Oligodendroglioma vs. IDH-wt Astrocytic Gliomas	1p/19q-Codel Oligodendroglioma vs. IDH-wt Astrocytic Gliomas; AUC	1p/19q-Codel Oligodendroglioma vs. IDH-mut Astrocytic Gliomas	1p/19q-Codel Oligodendroglioma vs. IDH-mut Astrocytic Gliomas; AUC
MK_0_	<0.0001	0.922	<0.0001	0.818	<0.001	0.799
MK_500_	<0.0001	0.869	<0.001	0.755	0.009	0.744
MD_0_	<0.0001	0.887	0.002	0.781	0.1	0.696
MD_500_	<0.0001	0.887	<0.001	0.790	<0.01	0.714

## Data Availability

All data related to this study can be provided by the authors upon request.
